# Prediction of Atypical Ductal Hyperplasia Upgrades Through a Machine Learning Approach to Reduce Unnecessary Surgical Excisions

**DOI:** 10.1200/CCI.18.00083

**Published:** 2018-12-18

**Authors:** Lia Harrington, Roberta diFlorio-Alexander, Katherine Trinh, Todd MacKenzie, Arief Suriawinata, Saeed Hassanpour

**Affiliations:** **Lia Harrington**, **Todd MacKenzie**, and **Saeed Hassanpour**, Geisel School of Medicine at Dartmouth College, Hanover; **Roberta diFlorio-Alexander, Katherine Trinh**, and **Arief Suriawinata**, Dartmouth-Hitchcock Medical Center, Lebanon, NH.

## Abstract

**Purpose:**

Surgical excision is currently recommended for all occurrences of atypical ductal hyperplasia (ADH) found on core needle biopsies for malignancy diagnoses and treatment of lesions. The excision of all ADH lesions may lead to overtreatment, which results in invasive surgeries for benign lesions in many women. A machine learning method to predict ADH upgrade may help clinicians and patients decide whether combined active surveillance and hormonal therapy is a reasonable alternative to surgical excision.

**Methods:**

The following six machine learning models were developed to predict ADH upgrade from core needle biopsy: gradient-boosting trees, random forest, radial support vector machine (SVM), weighted K-nearest neighbors (KNN), logistic elastic net, and logistic regression. The study cohort consisted of 128 lesions from 124 women at a tertiary academic care center in New Hampshire who had ADH on core needle biopsy and who underwent an associated surgical excision from 2011 to 2017.

**Results:**

The best-performing models were gradient-boosting trees (area under the curve [AUC], 68%; accuracy, 78%) and random forest (AUC, 67%; accuracy, 77%). The top five most important features that determined ADH upgrade were age at biopsy, lesion size, number of biopsies, needle gauge, and personal and family history of breast cancer. Using the random forest model, 98% of all malignancies would have been diagnosed through surgical biopsies, whereas 16% of unnecessary surgeries on benign lesions could have been avoided (ie, 87% sensitivity at 45% specificity).

**Conclusion:**

These results add to the growing body of support for machine learning models as useful aids for clinicians and patients in decisions about the clinical management of ADH.

## INTRODUCTION

Atypical ductal hyperplasia (ADH) is a high-risk breast lesion that confers approximately four- to five-fold risk of breast cancer.^1^ ADH is primarily detected via percutaneous core needle biopsy, during which multiple passes of the lesions are obtained to ensure proper sampling.^2^ However, only portions of the lesion are sampled; therefore, it is possible to miss cancerous tissue within the lesion.^3^ Biopsy modality (ultrasound, stereotactic, or magnetic resonance imaging guided) and needle size may influence sampling and accuracy such that the presence of cancer may be underestimated by 10% to 45%, depending on the percutaneous biopsy method used.^4^ Currently, the recommendation for diagnosis of ADH on core needle biopsy is surgical excision, because approximately 20% to 30% of these lesions are upgraded to cancer.^5,6^ As a result, 70% to 80% of women undergo a costly and invasive procedure for a benign high-risk lesion.

**Fig 1.**
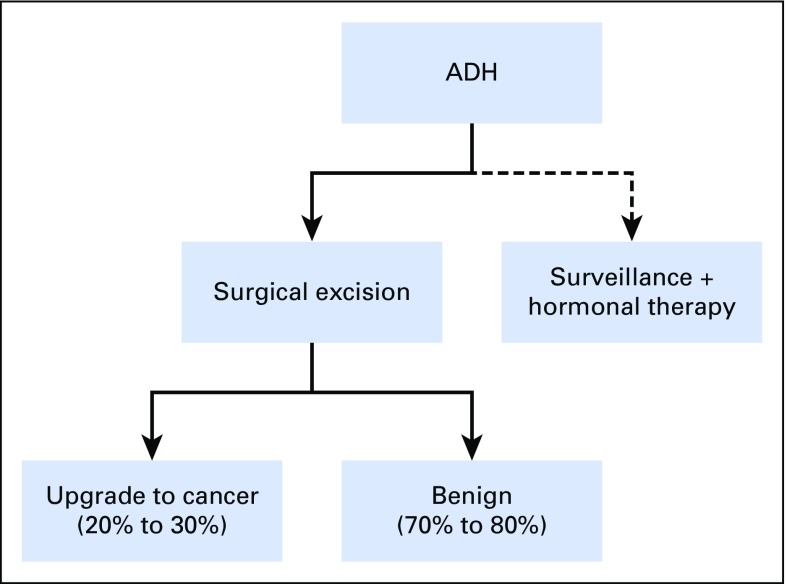
Flowchart of clinical management of atypical ductal hyperplasia (ADH) diagnosed on percutaneous core needle biopsy. The current clinical recommendation is surgical excision, but some women will opt for surveillance and hormonal therapy.

Surgical excision is more invasive than percutaneous core needle biopsy and has a higher risk of bleeding, infection, and postsurgical scar.^2,7^ The majority of ADH found on biopsies are not upgraded to malignancy, so identification of women at low risk of surgical upgrade would have a high clinical impact. For these women, active surveillance and possible chemoprevention may be a reasonable alternative.^8,9^ Figure 1 provides an overview of the current clinical management of ADH diagnosed by percutaneous core needle biopsy. A possible improvement compared with the current practice of surgical excision for all core needle biopsy–detected ADH may be that women at high risk of upgrade for surgical excision are targeted low-risk women are spared of potential overtreatment.

Previous studies have examined predictors of upgrade, such as biopsy needle gauge size,^10^ number of needle cores,^11^ number of foci of ADH/extent of ADH,^12^ presence of mass on mammography,^13^ size of lesions,^5^ and presence of calcifications.^14^ However, it is currently difficult to integrate these predictors into a clear recommendation criterion for surgical excision. An automated algorithm that precisely and quickly integrates this information into an accurate risk assessment would provide a clear benefit to clinicians and patients, who must make decisions about surgical excision. Thus, there is a critical need for a computational model that could learn the most informative features of lesions that are commonly upgraded to cancer, thereby possibly sparing low-risk women from invasive surgeries.

In pursuit of this, other groups have used various machine learning and computational approaches to predict such upgrade outcomes. Khoury et al^11^ created a nomogram using statistically significant features from a logistic regression model that had an area under the curve (AUC) of 77.5% to predict the likelihood of ADH upgrade. Some predictive features in the final nomogram included age, hormonal therapy use, number of involved cores, size of largest lesion, and presence of mass versus calcifications. Bendifallah et al^15^ developed a logistic regression model and a recursive portioning model with respective AUCs of 65% and 57% to predict ADH upgrades. According to this model, age older than 50 years, menopause onset, and lesion size greater than 10 mm were important to predict ADH upgrade. Using a multivariable logistic regression model, Peña et al^16^ found a low upgrade risk for women who had no individual cell necrosis, a single ADH focus with ≥ 50% removal, or 2 to 3 foci with ≥ 90% removal. They noted that occurrences that met the low-risk inclusion criteria had an upgrade rate of only 4.9% compared with 21.4% for those occurrences that did not. Most recently, Bahl et al^17^ achieved a sensitivity of 97.4% and a specificity of 30.6% using a random forest model to predict upgrade of all high-risk lesions, including ADH.

This study aimed to develop and evaluate six machine learning models to predict ADH upgrade from the core needle biopsy, which would potentially spare patients with benign lesions from invasive surgical excisions and maintain high sensitivity for prediction of malignant legions. The six chosen machine learning methods are gradient-boosting trees, random forest, radial support vector machine (SVM), weighted K-nearest neighbors (KNN), logistic elastic net, and logistic regression. Secondary aims of this study were to add to the growing body of literature in support of previously reported predictive machine learning features and to highlight new features that may predict upgrade of ADH diagnosed on core needle biopsy.

The key objective of this study was to show that a machine learning approach can spare women from unnecessary diagnostic excisions by computationally predicting whether ADH lesions are actually cancerous (ie, upgraded from initial core needle biopsy).

We developed a series of machine learning models that show promising performance for prediction of whether ADH lesions would be surgically diagnosed as cancer. A number of factors from medical records—including age, lesion size, number of biopsies, and history of breast cancer—were associated with lesion upgrade.

These models could reduce unnecessary surgeries by 16% (at 98% sensitivity), and they highlight what risk factors contribute most to upgrade of ADH lesions. They also demonstrate the promise of personalized medicine to tailor patient treatment according to the predicted risk of upgrade, and they show that different models come with important tradeoffs in sensitivity and specificity.

## METHODS

### Data Set

The study cohort consisted of the population of patients at Dartmouth-Hitchcock Medical Center with ADH identified by core needle biopsy and subsequent surgical excision from 2011 to 2017 to provide a definite upgrade status of the lesion. This cohort included 124 women who were 30 to 83 years old; four women had multiple lesions, which provided a total of 128 lesions in the data set. Thirty of these lesions were subsequently upgraded to cancer through surgical excision. ADH was considered upgraded if the surgical excision yielded ductal carcinoma in situ (DCIS) or invasive ductal carcinoma. Patients were excluded if they also had DCIS or invasive ductal carcinoma on the core needle biopsy. Patients with proliferative or benign lesions in addition to ADH were included, and these other conditions were included as features. This study and the use of human patient data in this project were approved by the Dartmouth institutional review board with a waiver of informed consent (STUDY00030375).

### Machine Learning Models

Data cleaning was performed in Python v2.7 (Python Software Foundation, Beaverton, OR). Machine learning and statistical analyses were performed in R v3.4.0 (R Foundation for Statistical Computing, Vienna, Austria) using the caret package.^18^ AUC curve plotting and analysis were performed using the pROC package for R software.^19^

#### Data preparation.

Categoric variables were separated into one feature per category, and missing categoric variables were left as not available. The cancer risk of women was encoded as (3) if the woman had cancer or any high-risk lesions previously (eg, ADH, DCIS, and lobular carcinoma in situ), as (2) if any cancer existed in the immediate family, as (1) if breast cancer existed in extended family, and as (0) if no cancer had been reported in the family. Missing continuous variables, such as age and size of lesion, were imputed with the overall mean values. Because scaling of features can sometimes improve model performance (depending on the model), numeric variables, including age, size of lesion, size of second lesion, and needle gauge, were mean-centered and scaled by their standard deviations.

#### Nested cross-validation.

Nested 10-fold cross-validation with hyperparameter grid selection was used to train six machine learning models and to estimate model performance. For each model, the corresponding caret method was used, as explained in each model’s description. Hyperparameter tuning was performed internally by caret for each model’s available set (Appendix Table A1), and the grid size was 5 for each parameter. For each model and combination of hyperparameters, the data were split into 10 partitions; nine of these partitions were used for training, and the tenth was partition held out to estimate model performance. Optimal hyperparameters were selected using the validation AUC.

#### Model evaluation.

**Fig 2.**
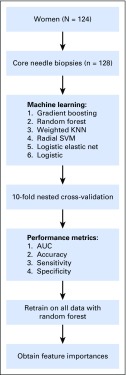
An overview of the training and testing methodology for our six machine learning models. AUC, area under the curve; KNN, K-nearest neighbors; SVM, support vector machine.

Using the selected combination of hyperparameters, 10-fold cross-validation was used to assess model performance under metrics of sensitivity, specificity, accuracy, and receiver operator characteristic AUC (a measure of overall model performance), including bootstrap confidence estimates with pROC.^19^ Model accuracy was determined at the default class probability threshold of 0.5. In addition, accuracy, sensitivity, and specificity of the model were determined by searching for an optimal probability threshold with a method similar to that of Song et al^20^ using the maximal geometric mean of sensitivity and specificity to select the attendant probability threshold. Secondary models trained on the entire data set were used to report random forest variable importance scores and logistic regression coefficients. An overview of the used machine learning models is shown in Figure 2.

#### Random forest.

A random forest model is an ensemble method that builds a set of decision trees that collectively make a robust and accurate prediction about an observation.^21^ A random forest is an improvement on a single decision tree, because each individual decision tree is a weak learner, but, when combined, they exhibit a stronger performance. For this analysis, the “rf” model in the caret package was used.

#### Gradient-boosting trees.

Gradient boosting is an ensemble machine learning model to form robust predictions on the basis of the integrated predictions of multiple simpler trees.^22^ Although each tree is a weak predictor of the outcome of interest, when combined, they can create an ensemble tree that is a good predictor. Each tree in gradient boosting is added such that it improves upon errors made by trees previously added to the ensemble. For this analysis, the “xgbTree” model in the caret package was used.

#### Gradient-boosting trees versus random forest.

Gradient-boosting trees and random forest mostly differ in how the individual trees in the ensemble predictor are grown and added. Gradient boosting grows simple trees that are specialized in prediction of attributes that may affect the outcome, whereas random forest grows complex trees with many features. The power of gradient boosting stems from stacking predictions of simple nonredundant trees so that each new tree learns from errors of the previous to predict the outcome, whereas the power of random forest stems from averaging out overly specific predictions from complex fully grown trees.

#### Weighted KNN.

KNN is a simple classification algorithm in which predictions of membership are made depending on the majority class of the *k* nearest neighbors. Weighted KNN improves performance of this classifier by incorporating the distance of the nearest neighbor, such that observations closer to the new observation are upweighted compared with more distant observations.^22^ For this analysis, the “knn” model in the caret package was used.

#### Radial SVM.

SVM models use a hyperplane to separate different classes while confidence margins in class predictions are maximized.^23^ The radial basis function kernel (RBF) was used as the function to separate classes. For this analysis, the “svmRadial” model in the caret package was used.

#### Logistic elastic net.

Logistic elastic net implements generalized linear models using maximum likelihood with elastic net penalization to find the predictors and associated weights that best predict the outcome. Lasso and ridge penalizations respectively imply use of L1 and L2 norms; elastic net penalization is a combination of L1 and L2 norms. These regularization schemes serve to downweigh predictors that do not improve model performance.^24^ Logistic elastic net selects which type of penalization is most optimal for the data. Here, “glmnet” in the caret package was used.

#### Logistic.

Logistic regression is a generalized linear model^25^ that uses a sigmoid function to transform the class scores to probability estimates. This comparatively simple model enjoys widespread use in clinical contexts, and its outcome can be interpreted as odds ratios. For this analysis, the “regLogistic” model in the caret package was used. This particular implementation included the regularization hyperparameters listed in Appendix Table A1. For simplicity and interpretability of the model, no higher-order interactions were considered.

### Statistical Analysis

95% CIs for AUC, sensitivity, specificity, and accuracy were determined using the pROC package^19^ with 2,000 bootstrap iterations. Statistical significance of associations between principal component axes with upgrade status was determined using the Wilcoxon-Mann-Whitney test.^26^

## RESULTS

**Fig 3.**
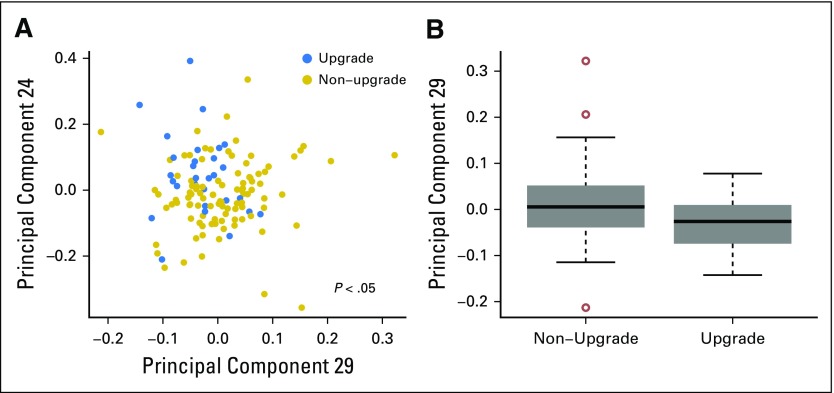
Principle coordinates weakly distinguish between upgraded and non-upgraded lesions. (A) Principal component plot of the two highest-associated principle components: PC29 (Wilcoxon-Mann-Whitney *P* = .01) and PC24 (Wilcoxon-Mann-Whitney *P* = .01). (B) Boxplot of highest-associated principle component, PC29.

Overall, 128 lesions from 124 women were collected for this analysis, of which 30 were upgraded. This study was concerned with discrimination between these two classes (upgraded *v* nonupgraded). When ordinated using principle component (PC) analysis, the lesions seemed to stratify by upgrade status on some of the axes with high statistical significance (PC29, *P* < .01; PC24, *P* < .01; PC32, *P* < .01; PC18, *P* < .05; all by Wilcoxon-Mann-Whitney test). The variables used in the ordination were a collection of 32 clinical variables that described the lesions. Depicted graphically, Figure 3 shows the two PCs (PC29 and PC24) that most significantly differentiated lesions by upgrade status. Visually, points that corresponded to upgraded lesions appeared nearer to the top left of the figure compared with the nonupgraded lesions, which provided additional indication that the two classes indeed differed, and it motivated more complex modeling in the form of supervised machine learning.

**Fig 4.**
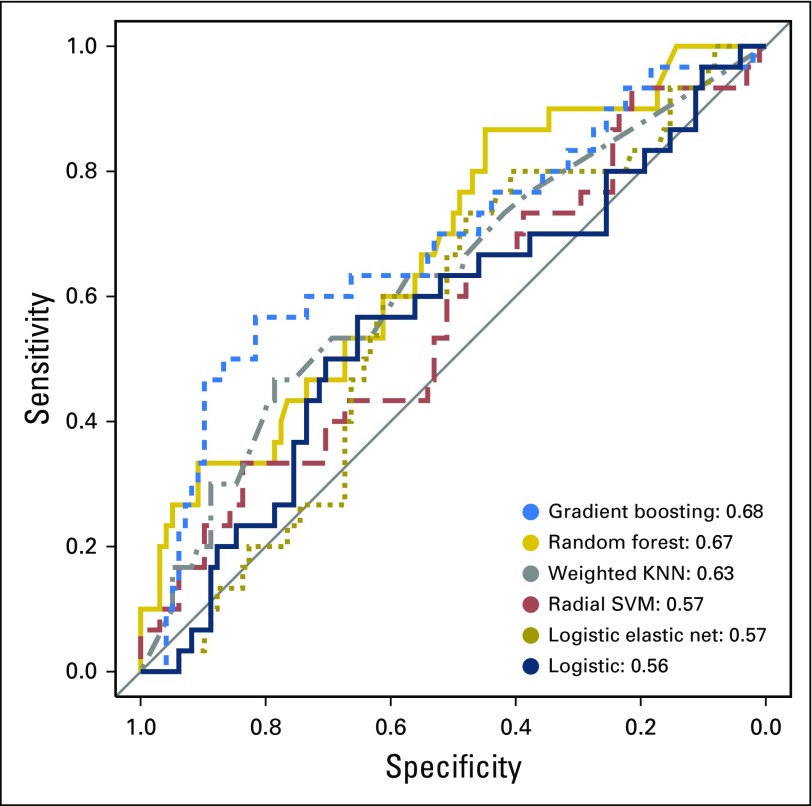
Receiver operator characteristic curves demonstrate differential classification efficacy of six classifiers on this data. Curves stretched toward the upper left of the plot indicate better model performance. Key is ordered by model accuracy using the area under curve (AUC) statistic and shows top performance by the gradient-boosting trees and random forest classifiers. KNN, K-nearest neighbors; SVM, support vector machine.

Accordingly, six machine learning models were trained to predict whether lesion biopsies would be upgraded to cancers given the same clinical variables. These models included gradient-boosting trees, random forest, weighted KNN, SVM with radial basis kernel, logistic elastic net, and logistic regression. The results of the models in terms of their performance are shown in Figure 4 and listed in Table 1. Gradient-boosting trees produced the highest AUC score, a measure of comparative model classification performance. The random forest model was a close second.

**Table 1.**
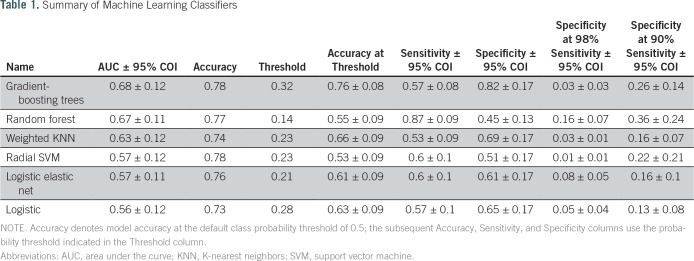
Summary of Machine Learning Classifiers

**Fig A1.**
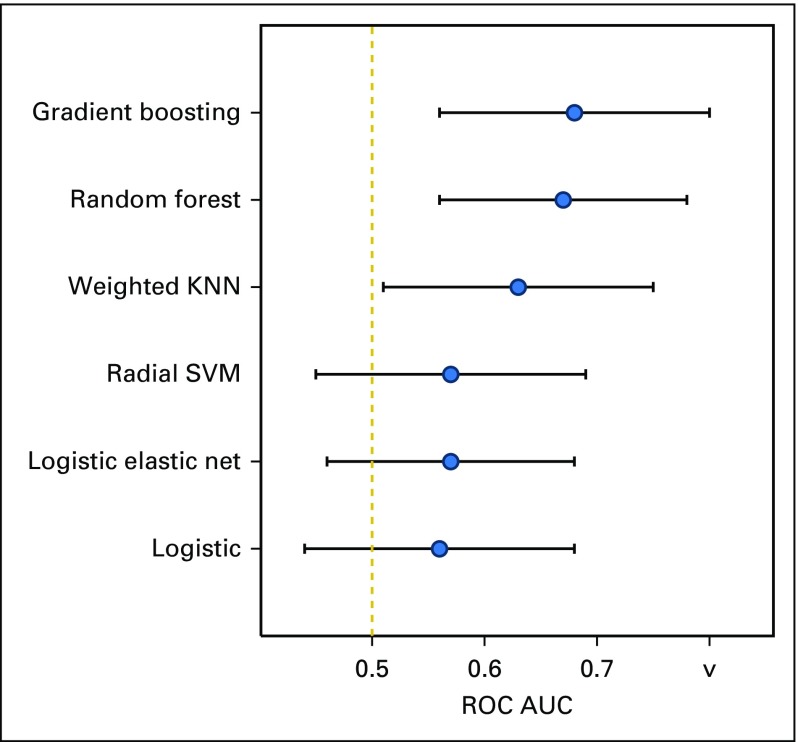
Comparison of model performance using the receiver operator characteristic (ROC) area-under-the-curve (AUC) metric. The gradient boosting trees, random forest, and weighted K-nearest neighbors (KNN) models deliver significantly better than random classification performance, as denoted by their 95% CIs, which did not cross the dotted red line at 0.5 AUC. The CIs were calculated by using 2,000 AUC bootstraps. SVM, support vector machine.

As listed in Table 1, the six models displayed different performance characteristics at selected sensitivity and specificity thresholds. Notably, the random forest model exhibited the highest specificity (0.16) at 0.98 sensitivity (a standard clinical cutoff) and 0.45 specificity at 0.87 sensitivity. To facilitate comparison, the model performances as reflected by AUC are also presented in Appendix Figure A1. Hyperparameters selected during cross-validation are reported in Appendix Table A1.

**Fig 5.**
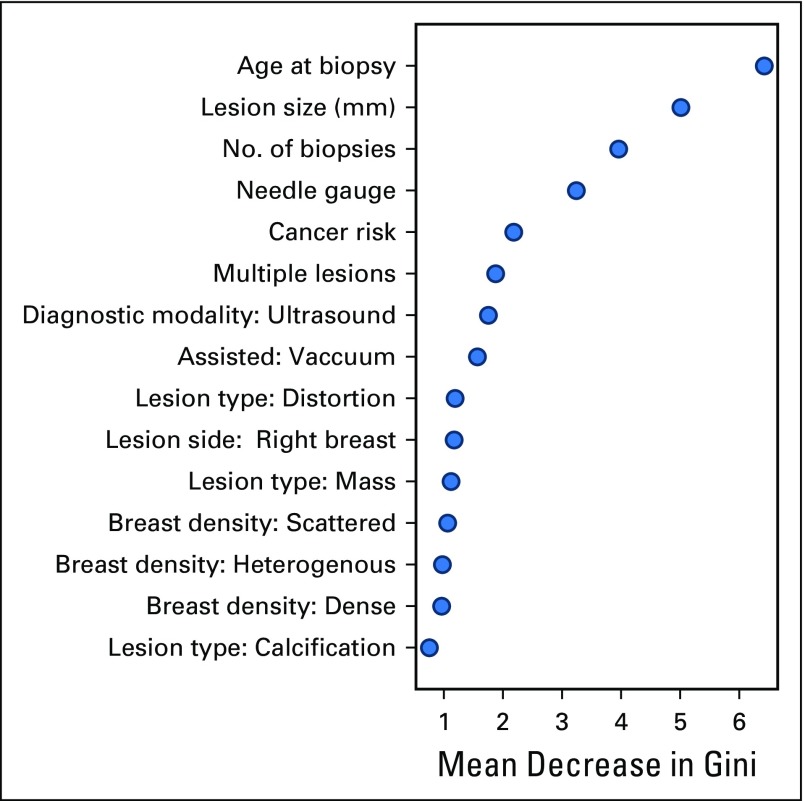
Variable importance scores reported by random forest classifier. The mean decrease in Gini coefficient coincides with the importance of that variable in making correct classifications in the random forest model. MRI, magnetic resonance imaging; N/A, not available.

**Fig A2.**
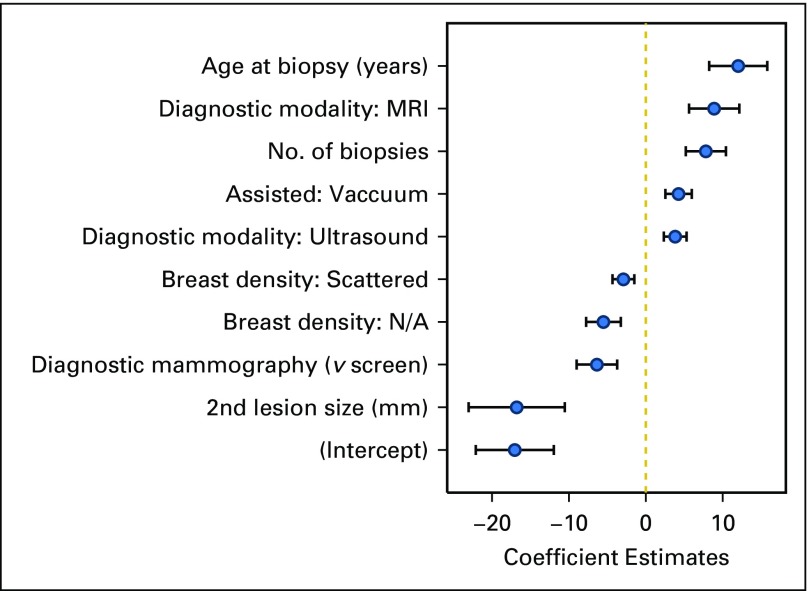
Logistic regression coefficients adjusted for all other variables for which *P* < .05. Error bars are standard errors of the mean. The red dotted line corresponds to noninformative coefficients (weight of zero), which were excluded from the plot. MRI, magnetic resonance imaging; N/A, not available.

The random forest variable importance scores are presented in Figure 5. Highly informative features for the model included age at biopsy, lesion size, number of biopsies, needle gauge, cancer risk, and the presence of multiple lesions. The regression coefficients for the logistic regression model, despite its lower AUC and overall performance, largely agreed with this importance ranking and are provided for reference in Appendix Figure A2.

## DISCUSSION

Current clinical guidelines recommend surgical excision for most cases of ADH detected by core needle biopsy.^27^ However, using a definitive surgical excision to rule out malignancy is not without harm. Because 20% to 30% of the ADH lesions are upgraded to DCIS or breast cancer at surgical excision, 70% to 80% of women undergo invasive surgical excision for benign atypical lesions. In this paper, we developed and compared the performance of six machine learning methods to predict ADH upgrade. Gradient boosting and random forests displayed 0.68 and 0.67 AUC scores, respectively. Although the gradient-boosting model performed slightly better, we focused on the random forest model here because of its comparative simplicity, interpretability, robustness, and well-characterized variable importance scoring.^28^ Interestingly, weighted KNN performed significantly better than a random model (which would have an AUC of 0.5) and ranked third in terms of predictive performance, behind gradient-boosting trees and random forest. Unfortunately, the bootstrap CI of the AUC for the logistic model (AUC, 0.56 ± 0.12 CI) included 0.50 (Appendix Fig A1), which indicates that its predictions were statistically indistinguishable from a random model. It is conceivable that consideration of higher-order interactions in the model would yield improved performance at the cost of increased model complexity.

As expected, age of patient at time of biopsy was the most important predictor of upgrade status. It is generally well known that cancer risk increases with patient age,^29^ and it is reassuring to see this trend confirmed in the model. The size of the lesion plays the second-largest predictive role, consistent with stochastically higher probability of cancer development and increased sampling error by core biopsy technique. Likewise, the number of biopsies, which played the third most important role in the model, may be explained by improved sampling and higher likelihood of inclusion of a malignant component of the lesion. Another high-ranking variable was personal and family history of cancer, which encapsulates the hereditary component of breast cancer, a factor that previously has been predictive for upgrade risk.^29^

This study only investigated high-risk ADH breast lesions. Although the narrow focus of the study allowed for a potentially stronger inference about this high-risk breast lesion, the inference does not cover other types of lesions. Furthermore, this study is based on data from a single, rural academic institution, and additional external validation is required to show the generalizability of our results. Thus, future work should focus on expansion of the scope of our model by inclusion of other high-risk breast lesions, such as lobular neoplasia, papillomas, and radial scars. In addition, these results could be extended by considering data from the New Hampshire Mammography Network,^30^ which includes all biopsies in New Hampshire, and other national breast cancer registries, which may not only improve this model but also better assess the generalizability of the approach. For future adoption of this approach, it is also important to model the risk of later progression to DCIS or malignancy if a benign lesion is kept in place using our method; previous studies suggest that roughly 20% to 30% of these lesions progress to DCIS or breast cancer.^31,32^

To conclude, the methodologic strength of this approach is based on rigorous training, testing, and comparison of six machine learning methods to predict ADH lesion upgrades. To the best of our knowledge, prior work has been limited to a small number of approaches for such a prediction task. Although the performance of the random forest model, given its AUC of 0.67, is lacking as a standalone clinical diagnostic tool, it nevertheless represents an important proof-of-concept and potential diagnostic aid. In addition, the results suggest that robust differences exist between low- and high-risk women and that machine learning models can reliably predict malignancy upgrade potential despite small sample sizes. This study also confirms important clinical variables involved in ADH upgrade risk. When our model is set at 98% sensitivity, 16% of surgical excisions of benign lesions would be unnecessary and could be avoided. Using the same model, if the target sensitivity is relaxed to 87%, 45% of lesions would be in the surveillance category. This relaxed sensitivity threshold, although associated with decreased sensitivity for cancer detection, may be useful as an exploratory aid to help patients and clinicians choose an alternative management approach. In this era of personalized medicine,^33^ such model flexibility may be desirable for patients who value a shared decision-making approach with the ability to choose between surgical excision for upgrade certainty versus surveillance to decrease unnecessary surgery.

## Figures and Tables

**Table A1.**
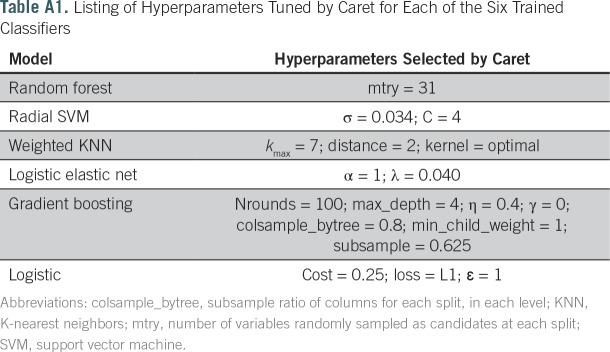
Listing of Hyperparameters Tuned by Caret for Each of the Six Trained Classifiers
